# Mortality rate in patients with symptomatic peripheral artery disease in Brazil: comparison between sexes

**DOI:** 10.31744/einstein_journal/2025AO1611

**Published:** 2025-09-08

**Authors:** Breno Quintella Farah, Raphael Mendes Ritti-Dias, Raquel Santana Fernandes, Antonio Eduardo Zeratti, Nelson Wolosker, Gabriel Grizzo Cucato, Marilia de Almeida Correia, Hélcio Kanegusuku

**Affiliations:** 1 Universidade Federal Rural de Pernambuco Recife PE Brazil Universidade Federal Rural de Pernambuco, Recife, PE, Brazil.; 2 Universidade Nove de Julho São Paulo SP Brazil Universidade Nove de Julho, São Paulo, SP, Brazil.; 3 Universidade de São Paulo Hospital das Clínicas Faculdade de Medicina São Paulo SP Brazil Hospital das Clínicas, Faculdade de Medicina, Universidade de São Paulo, São Paulo, SP, Brazil.; 4 Hospital Israelita Albert Einstein São Paulo SP Brazil Hospital Israelita Albert Einstein, São Paulo, SP, Brazil.; 5 Northumbria University Newcastle Upon Tyne UK Northumbria University, Newcastle Upon Tyne, UK.

**Keywords:** Peripheral artery disease, Hypertension, Cardiovascular diseases, Respiratory diseases, Intermittent claudication, Sex distribution, Mortality

## Abstract

**Objective:**

To compare mortality rates between sexes in a cohort of patients with intermittent claudication residing in a metropolitan city in Brazil.

**Methods:**

In this study, we included 215 patients (mean age 67±10 years, 65.3% men) who were followed for an average of 5.2 years (95% confidence interval [95%CI]: 4.8-5.5 years). At baseline, sociodemographic data, comorbidities, and clinical characteristics were recorded. The six-minute walk test was administered, with results reported as both absolute and relative walking distances, the latter based on reference values for healthy individuals with similar characteristics. Deaths were documented throughout the follow-up period. Cox regression analysis was used to estimate hazard ratios (HRs) and 95%CIs, adjusting for potential confounding factors.

**Results:**

A total of 105 patients (58.9% men) died, with cardiovascular diseases accounting for the leading cause of death (32.0%). Deceased patients were older, had a higher prevalence of hypertension, and demonstrated shorter absolute and relative walking distances. Men had a significantly higher risk of all-cause mortality compared to women, independent of age, chronic obstructive pulmonary disease, six-minute walking distance, and ankle-brachial index (HR: 2.774; 95%CI= 1.316-5.847).

**Conclusion:**

In patients with peripheral artery disease, men with intermittent claudication symptoms exhibit a higher risk of all-cause mortality compared to women. Future research should focus on identifying sex-specific risk factors associated with mortality in this population. Such insights are critical for developing targeted interventions aimed at reducing mortality, particularly among men with intermittent claudication in low- and middle-income countries.

## Highlights

■A total of 58.1% of the patients died during a mean follow-up period of approximately 5.2 years.

■Cardiovascular diseases are the main cause of death in these patients.

■Men had a higher likelihood of death from all causes than women.

■No difference was found between the sexes in relation to the different causes of death.

## In Brief

Of the 215 patients with intermittent claudication who completed a mean follow-up period of approximately 5.2 years, 105 died, with cardiovascular diseases being the main cause of death. Men had a higher likelihood of death from all causes than women (HR: 2.774; 95%CI=1.316-5.847).

## Graphical abstract

**Figure:**
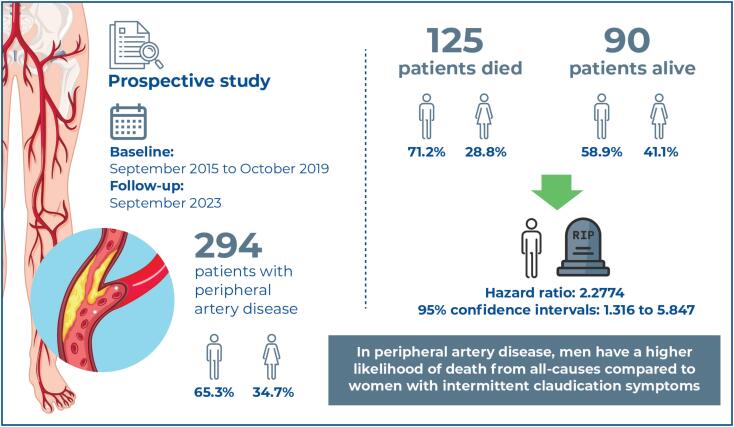

## INTRODUCTION

Peripheral artery disease (PAD) affects more than 200 million people worldwide, with a slightly higher prevalence in women than in men up to 75 years of age.^( 1 )^ The main symptom reported by these patients is intermittent claudication (IC), which is characterized by pain, cramping, or burning in the lower limbs during physical activity, and relieved by rest.^( 2 - 4 )^ Patients with IC experience walking impairments associated with several comorbid conditions,^( 5 , 6 )^ leading to an increased risk of mortality.^( 7 , 8 )^

Sex has been shown to be an important moderator of the consequences of PAD. Previous studies have observed that women with IC may experience more severe consequences of the disease, including worse walking impairment, impaired vascular function, and higher morbidity than men.^( 9 - 11 )^ However, with respect to mortality—a clinically relevant outcome—results have been inconsistent, with some studies reporting similar mortality rates between the sexes,^( 12 , 13 )^ and others indicating higher rates in women^( 14 , 15 )^ or in men^( 16 , 17 )^ with IC. The reasons for these discrepancies remain unclear. They may be related to the geographical locations where the studies were conducted, considering cultural differences, self-care practices, or healthcare systems. In this context, it is essential that interpretations take into account the country or region being studied. Further research is needed, particularly in Brazil, where no prior studies have been conducted.

Understanding such results could be useful in developing strategies for treating these individuals. Sex-based differences in mortality can help guide healthcare resource allocation. For instance, if women with PAD are found to have higher mortality rates than men, this could lead to the development of targeted cardiovascular programs for women and the implementation of health policies aimed at reducing such disparities. Moreover, identifying these disparities in large Brazilian cities could support awareness campaigns to educate healthcare professionals and the public on the importance of early diagnosis and sex-sensitive care for PAD. We hypothesized that women with PAD may have higher mortality rates than men due to greater disease severity.

## OBJECTIVE

In this study, we compare the mortality rates by sexes in a cohort of patients with intermittent claudication living in a metropolitan city in Brazil.

## METHODS

### Study design and participants

This prospective study included patients with IC recruited from the vascular units of two public hospitals in São Paulo, Brazil, between September 2015 and October 2019, with follow-up continuing until September 2023. The study was approved by the institutional ethics committee of the *Hospital Israelita Albert Einstein* (CAAE: 42379015.3.0000.0071; # 1.159.960). Prior to data collection, patients were informed about the study procedures and provided written informed consent.

Patients were eligible for inclusion if they met the following criteria: a) presence of IC symptoms (Fontaine stage 2A or 2B) in one or both legs;^( 18 )^ b) ankle-brachial index (ABI) ≤0.90; and c) absence of non-compressible vessels, limb amputations, and/or ulcers. Patients without contact information or with no available data in the official system were excluded from the analysis.

### Baseline information

Sociodemographic, comorbidity, and clinical data were obtained through face-to-face interviews using a previously described protocol.^( 19 )^ Briefly, data collected included smoking status (ex-, current-, or never-smoker), obesity (body mass index >30kg/m²), diabetes (physician-diagnosed or use of glucose-lowering medication), hypertension (physician-diagnosed or use of antihypertensives), dyslipidemia (physician-diagnosed or use of lipid-lowering medication), heart diseases (physician-diagnosed), other comorbidities (physician-diagnosed), and current medication use. Body weight was measured using a calibrated scale to the nearest 0.1kg, and height was measured with a stadiometer to the nearest 0.01m. Body mass index was calculated as weight divided by height squared (kg/m²), in accordance with a previously described protocol.^( 20 )^

Peripheral artery disease severity was assessed using the ABI, following established guidelines.^( 21 )^ The ABI was calculated as the ratio of the highest systolic blood pressure measured in the posterior tibial or dorsalis pedis artery to the highest systolic blood pressure in the brachial artery. Blood pressure measurements were taken in both limbs using a Doppler vascular monitor (DV160; Medmega, Brazil) and a sphygmomanometer, following a standardized protocol.^( 20 )^

Walking capacity was evaluated using the six-minute walk test conducted in a 30-meter corridor, as previously described.^( 22 )^ Two cones were placed 30 meters apart, and patients were instructed to walk as many laps around the cones as possible, reporting the onset of claudication symptoms (claudication onset distance). Total distance walked was defined as the maximum distance completed in six minutes. Relative total walking distances were estimated using normative data for healthy individuals with similar characteristics.^( 23 )^

### Mortality and follow up

Death events were identified through reports from individuals familiar with the patients, obtained via periodic telephone follow-ups conducted semi-annually or annually. In cases of lost contact, additional information was retrieved through specialized websites.

### Statistical analysis

All analyses were performed using IBM SPSS Statistics version 25. Continuous variables were reported as means (standard deviations), and categorical variables as frequencies. Normality was assessed using the Kolmogorov-Smirnov test. Differences in baseline characteristics by sex and survival status were evaluated using χ^2^ tests and independent sample *t* -tests. Effect sizes were calculated using Cohen's d for continuous variables and Phi (φ) for categorical variables to assess the magnitude of differences between groups.

Survival rates between men and women with PAD were compared using the Kaplan-Meier method, with survival curves analyzed using the Log-Rank, Breslow, and Tarone-Ware tests. Cox regression analysis was conducted to assess the association between sex and all-cause mortality, with hazard ratios (HRs) and 95% confidence intervals (95%CIs). The analysis was adjusted for chronic obstructive pulmonary disease, age, total walking distance, and ABI. Women served as the reference category. Statistical significance was set at p<0.05.

## RESULTS

**Figure 1:**
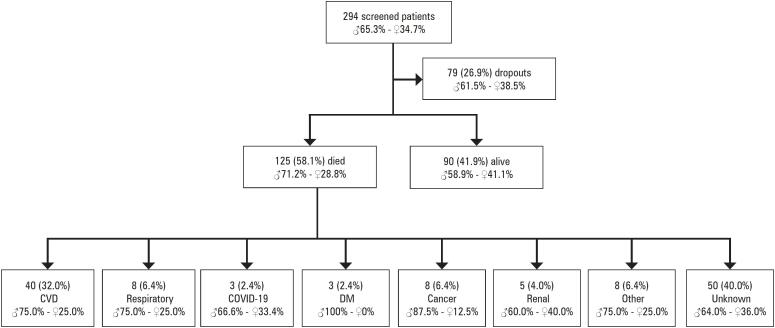
Causes of death among patients with intermittent claudication

CVD: cardiovascular diseases; DM: diabetes mellitus.

Initially, 294 patients were eligible for inclusion. However, some patients dropped out (n=79), resulting in a final sample size of 215 patients. The follow-up period was 5.2 years (95%CI= 4.8-5.5 years), during which 125 patients died (58.1%). Cardiovascular diseases, respiratory diseases, and cancer were the main causes of death in patients with IC ( Figure 1 ). No difference between the sexes was found in relation to the different causes of death (p>0.05).

Table 1 presents the patient characteristics according to sex. Women exhibited a shorter absolute total walking distance than men (p<0.05). Patient characteristics according to death status are shown in table 2 . Patients who died were older, had a higher prevalence of hypertension, and had shorter absolute and relative total walking distances (p<0.05).

**Table 1:** General sample characteristics of patients included in this study

Variables	All patients (n=215)	Women (n=73)	Men (n=142)	p value
Age (years)	67±10	66±10	67±9	0.197
Body mass index (kg/m^2^)	27.4±4.9	27.6±5.9	27.3±4.3	0.726
Total walking distance (m)	319±83	295±78	330±83	0.007
Relativized 6MWT (%)	59.2±15.2	58.4±15.4	59.6±15.0	0.634
Ankle-brachial index	0.57±0.18	0.58±0.19	0.57±0.17	0.719
Current smoker (%)	22.5	22.9	22.3	0.928
Diabetes (%)	53.8	52.9	54.3	0.855
Hypertension (%)	83.6	79.7	85.5	0.289
Dyslipidemia (%)	79.9	82.4	78.7	0.537
Heart failure (%)	13.3	9.4	15.3	0.256
Coronary artery disease (%)	37.8	36.4	38.5	0.767
Cancer (%)	12.7	7.4	15.4	0.122
COPD (%)	6.4	11.8	3.7	0.034
Renal insufficiency (%)	18.7	17.9	19.1	0.836
Stroke (%)	20.3	18.8	21.0	0.714

Values are presented mean±standard deviation or frequency. 6 MWT – 6-minute walk Test.

COPD: chronic obstructive pulmonary disease.

**Table 2:** Characteristics of patients who died (all cases) were compared with those who did not die at baseline

Variables	No death (n=90)	Death (n=125)	Effect size (d or Phi)	p value
Age (years)	64.8±9.9	68.0±9.0	0.34	0.014
Body mass index (kg/m^2^)	27.5±4.8	27.3±4.9	0.04	0.746
Total walking distance (m)	340±71	305±87	0.44	0.004
Relativized 6MWT (%)	63.3±13.0	56.5±16.0	0.46	0.003
Ankle-brachial index	0.59±0.18	0.56±0.18	0.17	0.374
Sex (% women)	28.8	41.1	0.128	0.060
Current smoker (%)	21.6	23.1	0.099	0.791
Diabetes (%)	48.3	57.9	0.095	0.172
Hypertension (%)	76.7	88.4	0.155	0.025
Dyslipidemia (%)	75.9	82.9	0.087	0.214
Heart failure (%)	7.3	17.7	0.151	0.053
Coronary artery disease (%)	35.3	39.7	0.044	0.529
Cancer (%)	9.2	15.4	0.092	0.190
COPD (%)	3.4	8.6	0.106	0.157
Renal insufficiency (%)	15.1	21.4	0.079	0.259
Stroke (%)	17.2	22.5	0.065	0.353

Values are presented mean±standard deviation or frequency. 6 MWT – 6-minute walk Test.

COPD: chronic obstructive pulmonary disease; d: Cohen.

**Figure 2:**
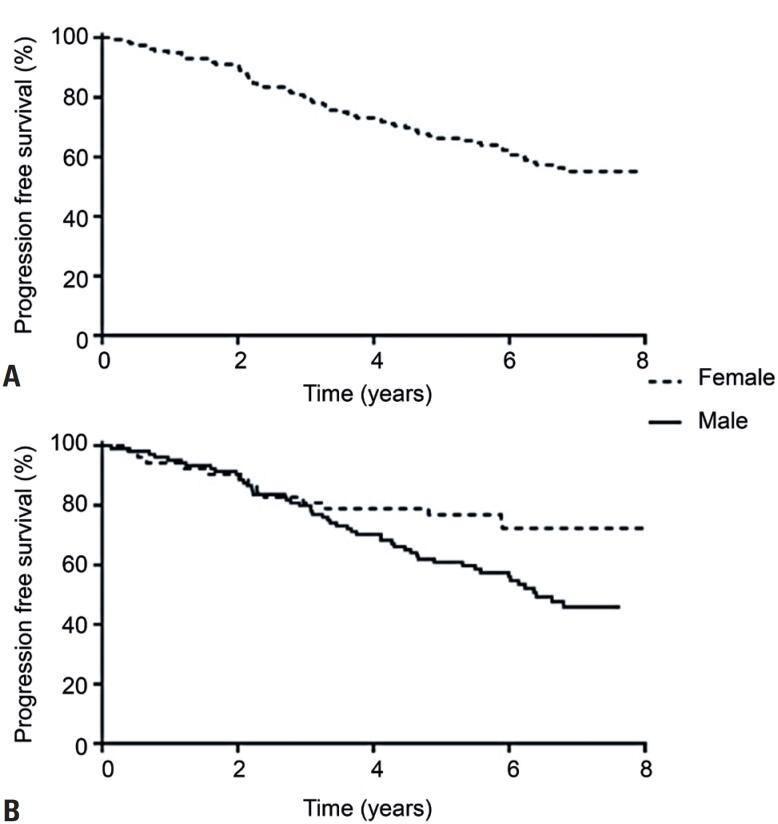
Survival curve in the 8 years follow-up for all 215 patients with symptomatic peripheral artery disease (Panel A). Survival curve in the 8 years follow-up in patients with symptomatic peripheral artery disease stratified by sex (Panel B)

Figure 2 presents the survival curve over an 8-year follow-up period for all patients with symptomatic PAD (Panel A) and stratified by sex (Panel B). A significant difference in mortality curves was observed between men and women, with men exhibiting lower survival rates. This was confirmed by the following statistical tests: log-rank test (p=0.013), Breslow test (p=0.047), and Tarone-Ware test (p=0.025), which indicated consistent results across various methods for comparing survival distributions.

The association between sex and all-cause mortality in patients with IC is shown in table 3 . Men had a higher likelihood of death from all causes than women, after adjusting for chronic obstructive pulmonary disease, age, total walking distance, and ABI.

**Table 3:** Crude and adjusted association between sex and all-cause mortality among patients with peripheral artery disease

Predictor	Category	Events	HR (95%CI) Crude	HR (95%CI) Adjusted
Sex	Women	36	Reference	Reference
	Men	89	2.084 (1.152 to 3.772)	2.774 (1.316 to 5.847)

Adjusted for chronic obstructive pulmonary disease, age, total walking distance, and ankle-brachial index.

HR: hazard ratio; 95%CI: 95% confidence interval.

## DISCUSSION

The main findings of the present study were as follows: 1) 58.1% of patients died, with cardiovascular diseases (32.0%), respiratory diseases (6.4%), and cancer (6.4%) being the leading causes of death among patients with IC; 2) patients who died were older, had a higher prevalence of hypertension, and demonstrated lower absolute and relative total walking distances; and 3) men with IC had a higher likelihood of all-cause mortality than women, independent of chronic obstructive pulmonary disease, age, total walking distance, and ABI.

To our knowledge, this is the first study to analyze mortality in patients with IC in Brazil. In this cohort, patients with IC showed a high mortality rate (58.1%). Individuals with PAD typically exhibit alterations in various cardiovascular parameters,^( 10 , 24 - 26 )^ such as elevated blood pressure, increased arterial stiffness, endothelial dysfunction, and impaired autonomic balance—all of which are well-established contributors to higher mortality. In the present study, cardiovascular diseases emerged as the leading cause of death, consistent with previous reports.^( 3 , 4 , 17 )^ However, it is important to note that non-cardiovascular causes of death were also common. These findings underscore the need to consider both cardiovascular and non-cardiovascular outcomes in the management of patients with PAD and IC.

Patients who died from all causes were older, had a higher prevalence of hypertension, and exhibited lower absolute and relative walking capacities. These factors are well known to be associated with poorer health outcomes and increased morbidity in patients with IC,^( 4 , 16 , 27 - 29 )^ which may explain the observed elevated mortality rates. These results support the need for strategies aimed at preventing and treating hypertension and improving functional capacity to promote survival in patients with IC.

Another notable finding was that one of the few differences in clinical profiles between sexes was the prevalence of chronic obstructive pulmonary disease, which was higher in women than in men. Additionally, while women had lower absolute walking distances than men, the relative total walking distance was similar across sexes, indicating that, when adjusted for age-matched healthy controls, these differences were no longer present. Therefore, the clinical profile alone does not appear to explain the higher likelihood of all-cause mortality observed in men. In this context, previous studies have shown inconsistent findings, with some reporting similar mortality rates between sexes,^( 12 , 13 )^ some indicating higher mortality in women,^( 14 , 15 )^ and others reporting higher mortality in men with IC.^( 16 , 17 )^ Liu et al.^( 16 )^ and Haine et al.^( 17 )^ both reported a higher likelihood of all-cause mortality in men with IC.

Interestingly, a previous study^( 30 )^ involving Brazilian older adults without PAD aged over 60 years found a similar trend. After a 2.5-year follow-up, men had a higher mortality rate than women (HR = 2.8; 95%CI= 1.9-4.2), a result that closely mirrors our findings (HR = 2.774; 95%CI= 1.316-5.847). These similarities suggest that the findings of the present study align with broader trends observed in Brazil and that the focus on patients with symptomatic PAD does not appear to affect the observed pattern of higher mortality in men. Nonetheless, future studies in Brazil should include mortality data from older adults without PAD to further explore this hypothesis.

One possible explanation for the higher mortality in men may relate to differences in health-seeking behaviors. Generally, women are more health-conscious, seek medical care more frequently, and are more likely to adopt lifestyle modifications.^( 31 , 32 )^ This behavioral trend may partially account for the higher mortality observed in men with IC. Data from Brazil's National Health Survey support this, showing that women utilize healthcare services more frequently than men.^( 33 )^ However, it is important to acknowledge that this factor was not assessed during the 5.2-year follow-up, which represents a limitation of the present study.

Other limitations include: 1) the cause of death could not be identified in 40% of cases, limiting the ability to fully assess PAD's impact on health outcomes and complicating the development of targeted health policies or interventions. Nonetheless, all-cause mortality data remain highly relevant, especially given the scarcity of such information in metropolitan Brazilian populations. Furthermore, similar data have been widely reported in international studies;^( 12 - 15 , 17 )^ 2) only patients classified under Fontaine stage II were included, limiting the generalizability of the results to other PAD stages; and 3) the absence of a control group without IC symptoms prevented us from determining the direct impact of symptoms on mortality. Future studies aiming to assess the role of IC in mortality should consider including asymptomatic patients for comparison.

## CONCLUSION

Among patients with intermittent claudication, men had a higher likelihood of all-cause death. Future studies should identify the risk factors for mortality in this group to support the development of strategies for reducing mortality in men with intermittent claudication.
